# A system for bedside assistance that integrates a robotic bed and a mobile manipulator

**DOI:** 10.1371/journal.pone.0221854

**Published:** 2019-10-16

**Authors:** Ariel S. Kapusta, Phillip M. Grice, Henry M. Clever, Yash Chitalia, Daehyung Park, Charles C. Kemp

**Affiliations:** Healthcare Robotics Lab, Institute for Robotics and Intelligent Machines, Georgia Institute of Technology, Atlanta, GA, United States of America; Università degli Studi di Perugia, ITALY

## Abstract

Various situations, such as injuries or long-term disabilities, can result in people receiving physical assistance while in bed. We present a robotic system for bedside assistance that consists of a robotic bed and a mobile manipulator (i.e., a wheeled robot with arms) that work together to provide better assistance. Many assistive tasks depend on moving with respect to the person’s body, and the complementary physical and perceptual capabilities of the two robots help with respect to this general goal. The system provides autonomy for common tasks, as well as an interface for direct teleoperation of the two robots. Autonomy handles coarse motions of the robots by estimating the person’s pose using a pressure sensing mat and then moving the robots to configurations optimized for the task. After completing these motions, the user is given fine control of the robots to complete the task. In an evaluation using a medical mannequin, we found that the robotic bed’s motion and perception each improved the assistive robotic system’s performance. The system achieved 100% success over 9 trials involving 3 tasks. Using the system with the bed movement or the body pose estimation capabilities turned off resulted in success in only 33% or 78% of the trials, respectively. We also evaluated our system with Henry Evans, a person with severe quadriplegia, in his home. In a formal test, Henry successfully used the bedside-assistance system to perform 3 different tasks, 5 times each, without any failures. Henry’s feedback on the system was positive regarding usefulness and ease of use, and he noted benefits of using our system over fully manual teleoperation. Overall, our results suggest that a robotic bed and a mobile manipulator can work collaboratively to provide effective personal assistance and that the combination of the two robots is beneficial.

## Introduction

Illnesses, injuries, long-term disabilities, and other situations can result in people receiving physical assistance while in bed. For example, patients in hospitals and people with severe disabilities living at home can spend substantial time in bed. Robotic systems can provide assistance to a user in bed using a robot arm fixed to a location (i.e., a fixed-base manipulator) [1], but this approach comes with challenges. The size of the bed and length of the reclined human body can make reaching task-relevant locations difficult for a fixed-base manipulator. A mobile manipulator has the advantage that it can move itself around the bed to better reach task-relevant locations [2–4]. Yet even with a mobile base, challenges remain. For example, people who spend substantial time in bed typically use bedding, such as sheets and blankets, as well as furniture, including nightstands and overbed tables, that can interfere with perception of the human body and reaching. We have previously suggested in a workshop paper that combining the capabilities of a mobile manipulator and a robotic bed might support improved bedside assistance [5]. The robotic bed can perceive the human body through sensors embedded on its surface, move the person’s body to task appropriate postures that are more accessible to the mobile manipulator, and even move itself out of the way of the mobile manipulator, such as by lifting itself up so the base of the mobile manipulator can slide underneath. In our workshop paper, we presented an initial prototype system with a preliminary evaluation. Here, we present a substantially improved bedside assistance system with a thorough evaluation of its capabilities, including the first evaluation of this type of system with a real user in a real environment.

Our bedside assistance system consists of Autobed, a custom robotic bed, and a PR2, which is a mobile manipulator that was produced commercially by Willow Garage. Autobed and the PR2 collaborate to assist with tasks around the user’s body. To manage the system’s complexity for common assistive tasks, the system provides autonomous functions with a coarse-to-fine approach. It autonomously completes the “coarse” parts of the task, such as moving the mobile robot’s base to an appropriate place and configuring the robotic bed, and then gives control back to the user for “fine” execution, the detailed performance of the task. In our previous work in which users teleoperated a system without autonomous functions, Grice et al. [6] observed that a significant amount of the time that the user spent on each task was dedicated to moving the mobile robot into an appropriate position and moving the robot’s gripper near the task area. This “coarse” setup can be challenging and requires the user’s attention and effort. The system we present in this paper can reduce the user’s overall workload by having the system perform this part of a task autonomously. Once the robotic bed and the mobile manipulator are in position, the system transfers control to the user. This can be desirable, since the “fine” parts of the task can be difficult to automate due to challenges such as perception in clutter, dexterous manipulation near the human body, and dependence on information to which only the user has direct access. For example, in a scratching task, the location of an itch and how to satisfactorily scratch it would only be directly evident to the user receiving assistance.

When a user commands our system to assist with a task, the system creates and executes a plan. The plan typically begins with the mobile robot finding the relative location of the bed. Then, the robotic bed estimates the position of the user’s body using a pressure sensing mat and configures itself to position the person’s body for the task. The mobile manipulator moves to a position with respect to the person’s body from which it can reach task-relevant areas. Then, the manipulator reaches out autonomously to the task area if it can do so easily and safely. Specifically, it reaches out when the task area is not by a sensitive region like the person’s face, and when the task area is clear of sensitive obstacles like a user’s laptop used to control the robot. At this point the autonomous function ends and the user takes control of the robot via a web-based interface for teleoperation of the mobile manipulator and the robotic bed.

**Fig 1:**
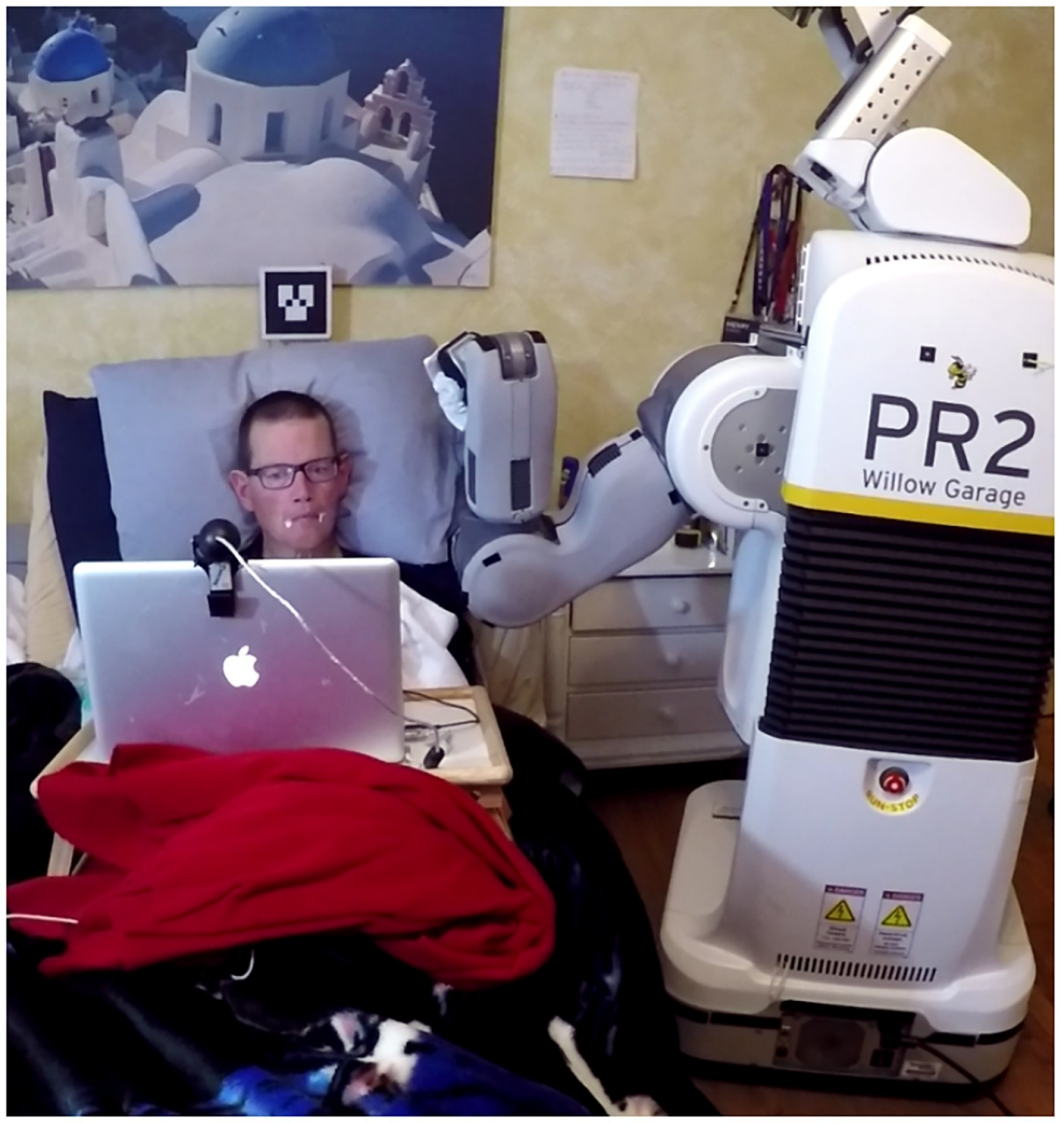
The system in use in a person’s home. Henry Evans, a person with severe quadriplegia, used our system in his own home to wipe yogurt from his mouth, visible as white dots on his face.

With this system, we investigated the potential for a robotic bed to collaborate with a mobile manipulator in order to provide more effective assistance to people in bed. Dental hygienists, barbers, and other professionals who perform tasks around the human body sometimes position peoples’ bodies using adjustable furniture. By doing so, the professional can improve ergonomics and the quality of the services they perform. The two robots in our system coordinate in an analogous manner. We demonstrate that a robotic bed and a mobile manipulator have complementary physical and perceptual capabilities. The robotic bed can move the human body using a small number of degrees of freedom, while the mobile manipulator can dexterously manipulate a lightweight payload using a larger number of degrees of freedom. The robotic bed can also perceive the human body via a pressure sensing mat, while the mobile manipulator would typically perceive the person using on-board line-of-sight sensors that can be obstructed by bedding and other objects around a bed. Fig 1 shows the system in use at a person’s home as part of our evaluation.

We discuss related work in the next section. We then describe the system implementation (System Description). After which, we present the three evaluations we performed (Evaluation). We conducted this research with approval from the Georgia Institute of Technology Institutional Review Board (IRB), and obtained written informed consent from all participants. We then present the results from our three evaluations (Results), a discussion of our findings (Discussion), and our conclusions (Conclusion).

## Related work

Although we are unaware of other efforts that have integrated a robotic bed and a mobile manipulator, there are precedents in the literature for aspects of our system for bedside assistance, which we highlight in this section. We also cite illustrative examples from a number of research areas to which our system relates.

### Assistive robotics

Researchers have long explored the idea of assistive robots, especially for people with motor impairments. In some earlier work, researchers have developed desk-mounted assistive robots, such as the Handy rehabilitation robot by Topping and Smith [7]. Vijay et al. present a review of assistive robotic devices in the literature in 1997. Brose et al. present a later review of the advances in assistive robotics up to 2010 [8]. Some other recent work has investigated assistive robots fixed to desks, wheelchairs, or other stationary points [9, 10]. Bilyea et al. present a review of recent works on robots for personal care and assistance [11].

### Assistive mobile manipulation

Researchers have investigated the use of mobile manipulators as assistive devices, such as in [12–19]. Some researchers have explored smart home concepts that can include multiple robot or smart devices to assist people with motor impairments [20–22]. Park et al. presented a smart home that uses multiple robot agents, including mobile manipulators, to assist people with motor impairments around the home [23].

Some work has focused on methods for general-purpose mobile manipulators to assist with specific assistive tasks [24, 25]. A few tasks that have been investigated are household cleaning [26], generic in-home mobile manipulation [27, 28], shaving [29], feeding [30], picking-and-placing tasks [31], and transfers [32]. Some groups have looked at mobile manipulators providing assistance to users in bed. Cavallo et al. presented a robotic home assistance system using a mobile manipulator collaborating with smart devices around the home. Their smart devices assisted the robot with monitoring the environment and with localization [4].

### Collaborative robots

Many groups have explored multi-robot, collaborative systems [33–36], including heterogeneous multi-robot systems [37–39]. Some work on assistive robotics makes use of multi-robot systems [4, 23]. Additionally, some commercial surgical robots feature collaboration between heterogeneous robots, such as robotic surgical arms and a robotic surgical table [40, 41]. Our approach is similar, applying the idea of heterogeneous multi-robot collaboration to the field of assistive robotics.

### Robotic beds for physical assistance

Several groups have developed robotic beds that assist with or prompt a user to roll over using rollers or actuators parallel to the longitudinal axes of the body [42, 43]. Others have constructed novel robotic beds with greater control of the position of the body [44, 45] or with robotic mechanisms to assist the person in position themself [46]. Some modern robotic beds are commercially available [47]. Bilal et al. present a review on assistive technologies for users in bed [48]. In this work, we have used a robotic hospital bed named Autobed [49] as an agent in a collaborative heterogeneous multi-robot system.

### Body pose estimation in bed

Perceiving the body of a person in bed presents a challenge for robotic assistance with activities of daily living (ADLs), especially since the body can be occluded by bedding and other objects, such as furniture and medical equipment. It is an active area of research with some groups using various sensor modalities to estimate the pose, such as RGB video [50] or depth images [51].

Pressure sensors placed on the bed can provide a sensor modality that is unaffected by visual occlusions. Many researchers have used pressure images from such pressure sensors to estimate the person’s pose by fitting geometric or kinematic models to the images [52–54], by using direct clustering and classification-based methods [55], or using convolutional neural networks [56, 57]. In this work, we have fit a geometric and kinematic model based on an estimated center-of-mass from pressure sensor data.

### Selecting robot configurations for mobile manipulation

Prior research has investigated how to find good configurations for a mobile robot. A common method is to address the problem using inverse-kinematics (IK) solvers [58, 59]. Often, sampling-based methods are used to find robot base poses that have valid IK solutions, often as part of motion planning [60–63]. A group of work has been based on the capability map presented by Zacharias et al. [64], such as [65–67]. Other methods to select configurations for robots include using data-driven simulation [68, 69] and inverse-reachability maps [70–72]. In our work, we use task-centric optimization of robot configurations (TOC), a method from [3] to select one or two robot configurations for the mobile manipulator and robotic bed to perform each task. TOC explicitly models features and parameters of the environment, user, and task, that may be important to task performance. TOC implicitly handles error, jointly optimizes the configurations of the bed and mobile manipulator, and can be used to select one or two configurations of the two robots.

### Our previous work

We described an earlier version of our bedside-assistance system in a workshop paper [5]. Our current system differs substantially. It incorporates improvements to the web-based interface, a planning domain definition language (PDDL) framework for assistive-task planning, modeling of the user’s physical capabilities, a new body position estimation method, and user customization (including modeling the user’s body size and his or her human-computer interface). For the current paper, we conducted entirely new evaluations, including improved evaluations based on [5] and the first evaluation of our system in a real home with a person with disabilities.

Our bedside-assistance system incorporates a number of components described in previous publications [3, 6, 28]. [3] presents the method for selecting robot configurations for a task, but only presents results from computer simulations and did not compare performance with and without collaboration between a robotic bed and a mobile manipulator. Neither [28] nor [6] considers coordination between a robotic bed and a mobile manipulator. [28] presents the basic teleoperation interface for the PR2 robot, but incorporates only low-level autonomy. [6] is a workshop paper on the teleoperation system for the PR2 robot that includes a description of the planning framework and autonomy for picking and placing objects.

## System description

Within this section, we provide pertinent details about the main features and components of the bedside assistance system.

### Collaboration

**Fig 2:**
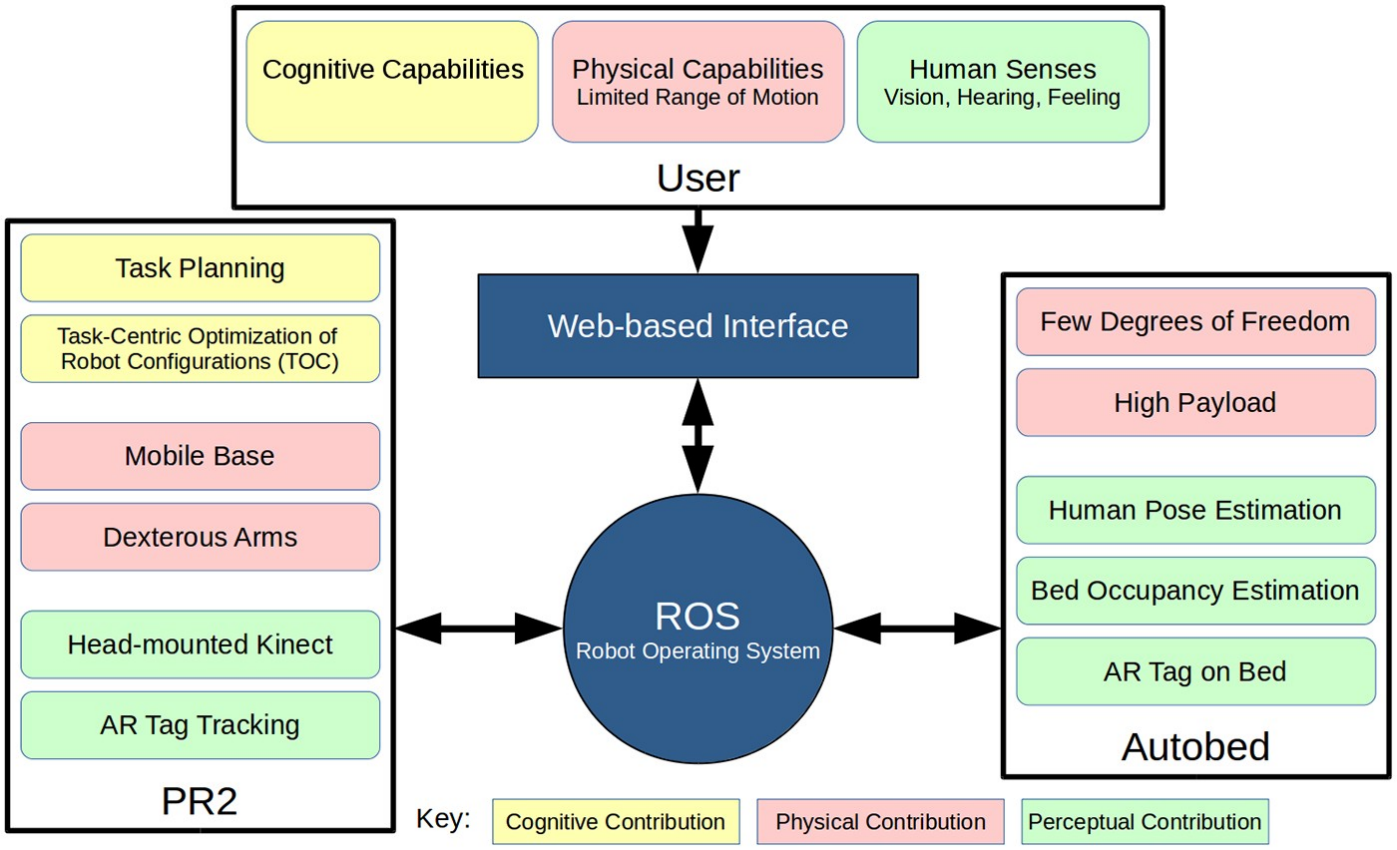
System architecture. Shows contributions of each component.

As illustrated in Fig 2, our system integrates a number of components. It uses the collaboration between two robots, a PR2 and Autobed, and a human user, leveraging the strengths of each. We focus on the cognitive, physical, and perceptual collaboration between the three.

With respect to cognitive collaboration, the system leverages the user’s cognitive capabilities to provide high-level direction and oversight during the autonomous setup for the task. The autonomous setup relies on the user to ensure that the system begins in an appropriate state. For example, the person should be lying on the bed in the correct direction. The PR2 generates and executes a high-level task plan, and identifies an optimized configuration of the PR2 and robotic bed (and therefore user) for completing the task. The user also provides control input for the fine control to perform the task, which may be difficult to complete autonomously. Although the entire task could be performed by having the user teleoperate the mobile manipulator and the robotic bed, this can be challenging. For example, Hawkins et al. observed that some assistive tasks require that a mobile manipulator use multiple base positions, and that manually choosing positions for some tasks can be difficult [29]. These autonomous functions allow the user to offload parts of the task, only requiring the user to oversee correct operation, thus reducing cognitive load. The goal of the autonomous functions is to configure the robots such that the task can be performed without further movement of Autobed or the PR2’s mobile base.

With regard to physical collaboration, Autobed has few degrees of freedom and an immobile base, but it has a high payload capacity, allowing it to position itself and the body of the user. The PR2 has a mobile base, high dexterity, and many degrees of freedom, but a low payload capacity. This allows the PR2 to move a lightweight tool to task-relevant locations around the user. Although the human user’s motor impairments may limit certain motions, they may have high dexterity within those limits, allowing them to perform the subtle motions necessary for task performance once the bed and PR2 have appropriately positioned a tool. For example, in trials with Henry Evans, Henry often moved his face against the towel held by the robot to wipe his face.

**Fig 3:**
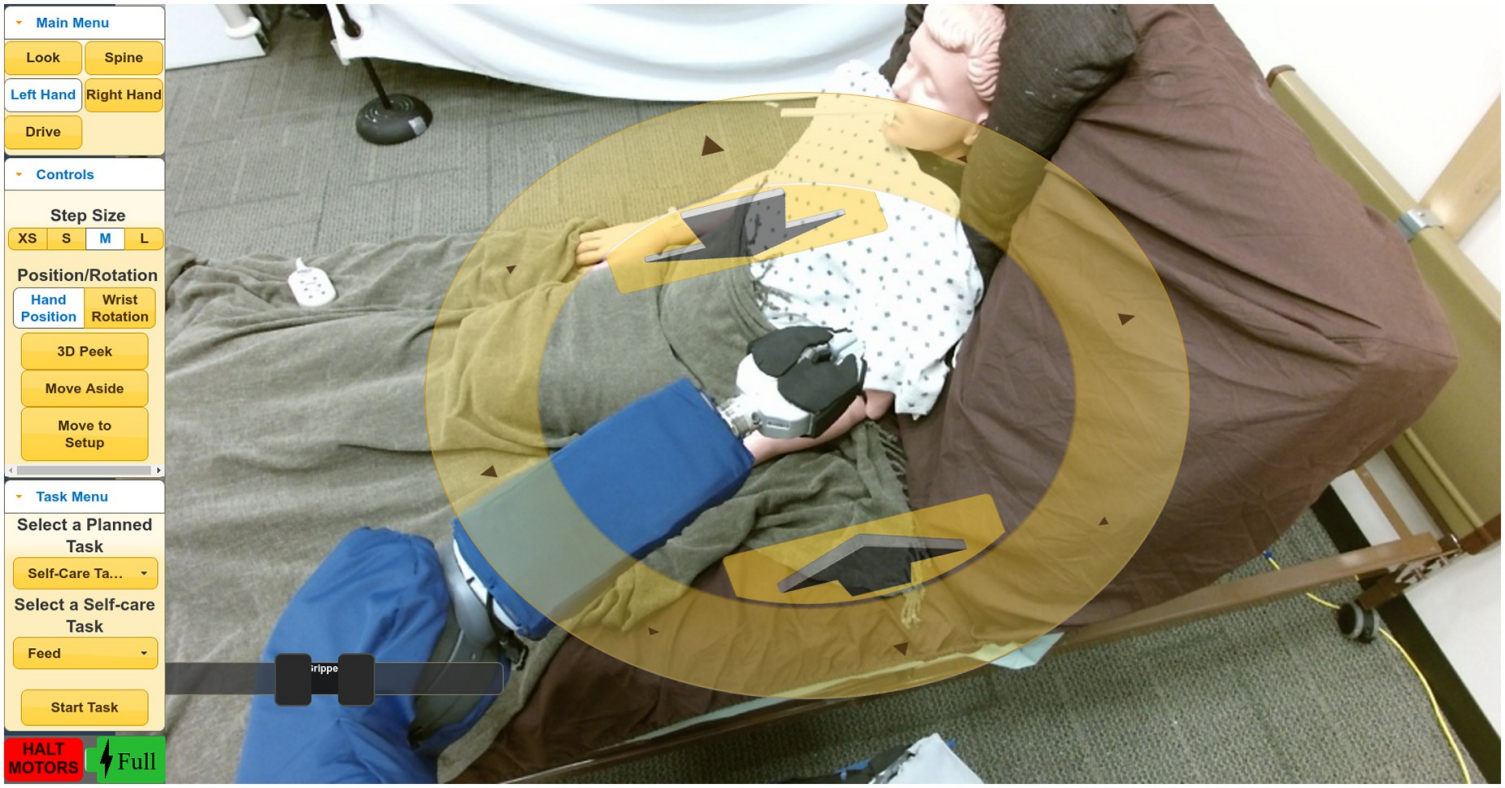
The web-based user interface. This interface enables low-level teleoperation by the user as well as the selection of autonomous planned tasks. Control modes and autonomous functions can be selected via buttons on the left.

With respect to perceptual collaboration, Autobed’s joint encoders and pressure-sensing mat estimate the position and configuration of the bed and the human on the bed. The PR2’s head-mounted Kinect v2 RGB-D camera visually locates Autobed via an ARTag. The user supplements their own senses with views provided by the PR2’s Kinect through the web-based interface shown in Fig 3 and described in [6, 28], which allows for low-level teleoperation and directing autonomous execution of planned tasks.

### The robotic bed (Autobed)

Autobed is a custom robotic bed based on a commercial actuated hospital bed (Invacare Full-Electric Homecare Bed, product ID: 5410IVC). In [49], we presented an earlier version of Autobed that provided web-based control of the bed’s movements. For the autonomous system we describe here, we used a newer version of Autobed that uses the Robot Operating System (ROS) and has sensors, actuators, geometric and kinematic models of itself, and autonomous control. The bed has 3 degrees of freedom: it can adjust its height, the angle of its backrest, and the angle of the leg rest. However, Autobed did not control the leg rest for the work we describe in this paper.

#### Autobed control

In order to enable Autobed to move, we added custom hardware between the bed’s standard remote control and the bed’s motor drivers. We used a Raspberry Pi single-board computer to interface with the bed’s actuators and we run the other Autobed functions on an adjacent computer.

Autobed uses accelerometers to measure the angle of the bed’s backrest with respect to gravity. We mounted a Hokuyo laser scanner to the underside of Autobed, pointing towards the floor to measure the height of the bed. Autobed fits a line to the scanner’s measurements using RANSAC to produce a height estimate robust to some obstructions (e.g., a foot). Autobed’s actuators are capable of changing the height of the bed and the angle of the backrest when a human is lying on the mattress. Autobed runs a simple bang–bang controller with a deadband to reach commanded configurations.

#### Human pose estimation

We have equipped Autobed with a pressure-sensing mat, manufactured by Boditrak (http://www.boditrak.com/ Model # BT3510), to measure the pressure distribution of the person lying on the bed. We placed the pressure-sensing mat on the top side of the mattress and below a fitted sheet. The pressure-sensing mat returns a pressure value for each of its 64 × 27 tactile pixels (taxels) at 5Hz. Autobed sums the pressure values to estimate the total weight on the pressure-sensing mat. When this estimate exceeds a threshold (we used ∼20 kg), it reports that the bed is occupied.

**Fig 4:**
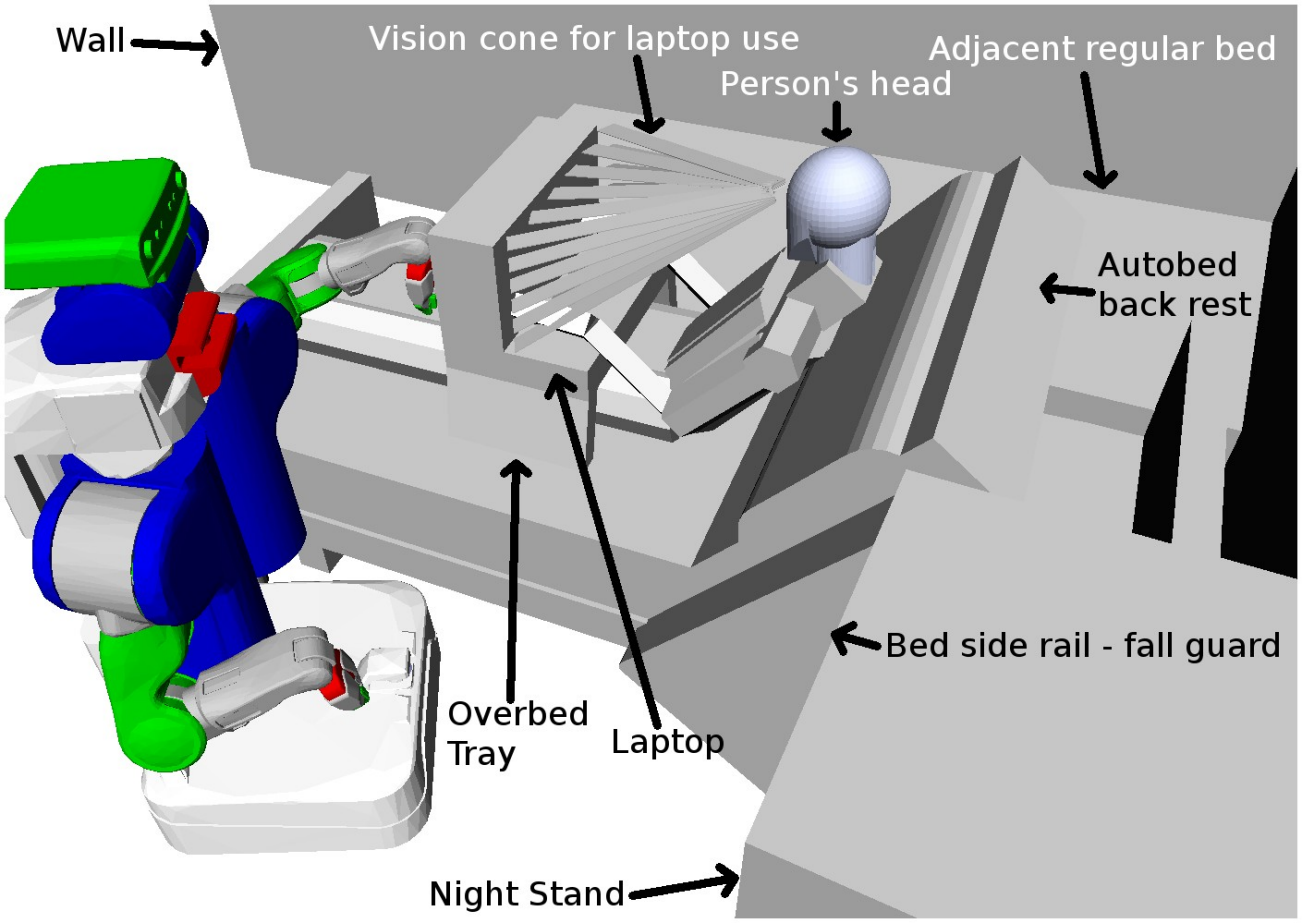
Autobed and the estimated pose of a person in bed. Left: View of a participant wearing various infrared reflective markers lying on Autobed. Right: Visualization of the pressure-sensing mat measurements from the participant with the estimated head position marked by a star inside a circle. The position in the Y direction is estimated from the pressure map. The position in the X direction is assumed to be ∼25 cm from the top of the mat.

Autobed uses a center of mass estimator from scikit-image (http://scikit-image.org/) to estimate the position of the body on the pressure-sensing mat. On average, the human pose estimation algorithm takes <1 millisecond to run on an external machine (Intel Core i7-3770, 3.40GHz). Fig 4 shows the estimated head position for a person lying on Autobed.

Autobed sets the Y-coordinate (the Y-axis is along the width of bed) of the body model’s pose equal to the Y-coordinate of the estimated center of mass estimate. Autobed positions the head of the body model in the X-direction to be ∼25 cm from the head of the bed and orients the body model such that the midline of the body model is parallel to the centerline of the bed.

**Fig 5:**
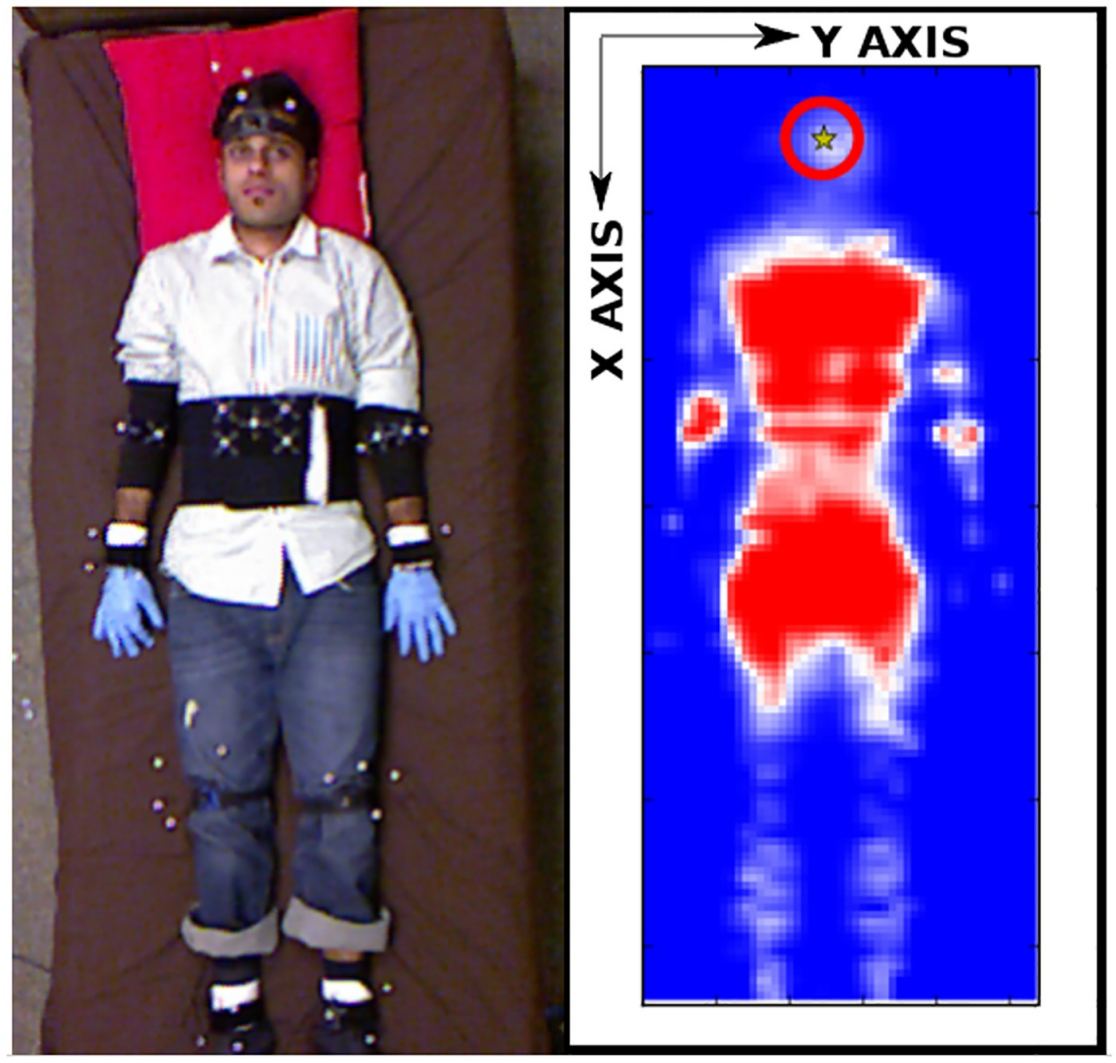
The customized model of Henry’s bedroom used by the system. This is the simulation environment used by TOC, our system’s configuration selection method. An example of customization is the inclusion of rays from the human model’s eyes to the laptop screen, so that Henry would be able to see his laptop throughout the task.

We constructed an approximate model of a supine human body from geometric primitives in the Unified Robot Description Format (URDF) file format, which allows the system to be customized by scaling the model from a 50th percentile male to specific users using Xacro. Xacro stands for XML Macros, a language with which URDF files can be generated from macro’ed properties. Fig 5 shows the simulation environment with an example bed and human configuration. Coordinate frames associated with the model’s body parts (e.g., knees, feet) are included in the model, although those parts are not visibly distinguishable.

In our workshop paper, our system estimated the position of the head using a blob detector on the pressure image [5]. Subsequently, we found that at higher angles of the bed’s backrest, the head’s visibility was significantly reduced in the pressure images. Using the estimated center of mass of the body to position the body along the Y axis and assuming a position along the X-axis avoids this issue and worked in our evaluations.

### The mobile manipulator (a PR2)

Our system uses a PR2 robot, a general-purpose mobile manipulator from Willow Garage. The PR2 has a mobile base, two 7-degree-of-freedom (DoF) arms with grippers, a pan-tilt (2-DoF) head, and a head-mounted Kinect v2. The arms have high dexterity, but a low payload capacity of 1.8 kg.

#### PR2 mobile base servoing

The system uses the ARTag servoing from [29] to move the PR2 mobile base directly from its current position to a goal position and orientation defined with respect to Autobed’s ARTag. This is accomplished via straight-line path and assumes that there are no obstacles to block PR2 movement. Our system uses the code package ar_track_alvar (http://wiki.ros.org/ar_track_alvar) to track an ARTag mounted on the headboard of Autobed, and thereby to locate Autobed. Fig 1 shows the ARTag as mounted on the bed. The PR2 moves its head to keep the ARTag in the center of its head-mounted Kinect’s view.

#### Haptic MPC control of the arm

Our system uses a newer version of the model predictive controller (MPC) described in [2], with low stiffness in both manual control mode and autonomous functions, to move the PR2’s arm to end effector poses (position and orientation) or joint configurations (when resetting the arm configuration between trials). Note that our current system does not use the fabric-based skin or tactile sensing from the original work.

### Optimization of robot configurations

In order for the system to autonomously move to an appropriate configuration for a task, the system first selects the configuration of the PR2 and Autobed using Task-centric Optimization of robot Configurations (TOC). TOC is based on [3]. TOC performs substantial offline computation to generate a function that can be applied rapidly online to select robot configurations based on the current user’s body size and observations of the user’s position on the bed. Fig 5 shows the simulation environment used by TOC, demonstrating a configuration for the *blanket adjustment* task.

Offline, for each task, TOC jointly optimizes one or two 6-DoF system configurations, each of which consists of a 4-DoF configuration for the PR2 (X-Y base position, base orientation, and Z-axis height) and a 2-DoF configuration for Autobed (Z-axis height and backrest angle). TOC models each task as a sparse set of 6-DoF end-effector goal poses. TOC runs the optimization for samples of the person’s position on the bed, given robot, person, and environment models, and then it interprets the optimization results to see if a single configuration of the two robots is sufficient for the task, or if there is value in using two configurations. Online, TOC applies the generated function for the desired task and current observations to inform the system what configurations to use for the task.

The task models used by TOC can be customized for a particular user and environment. TOC models the user’s physical capabilities (e.g., can rotate head up to 60 degrees), the user’s human-computer interface (e.g., a laptop, a head-tracker, and virtual obstacles to prevent the robot from interfering with their use), and important features of the user’s environment (e.g., a nightstand by the bed). TOC also uses a human model of similar size to the user. These customizations allow TOC to better select robot configurations.

### Task planning

The system uses the task-level planning system from [6], based on the PDDL [73]. When possible, the planner produces a correct, minimal sequence of actions to complete the task. This sequence is then used to produce and execute a Hierarchal Finite State Machine (HFSM), implemented using the ROS SMACH framework (http://wiki.ros.org/smach).

**Fig 6:**
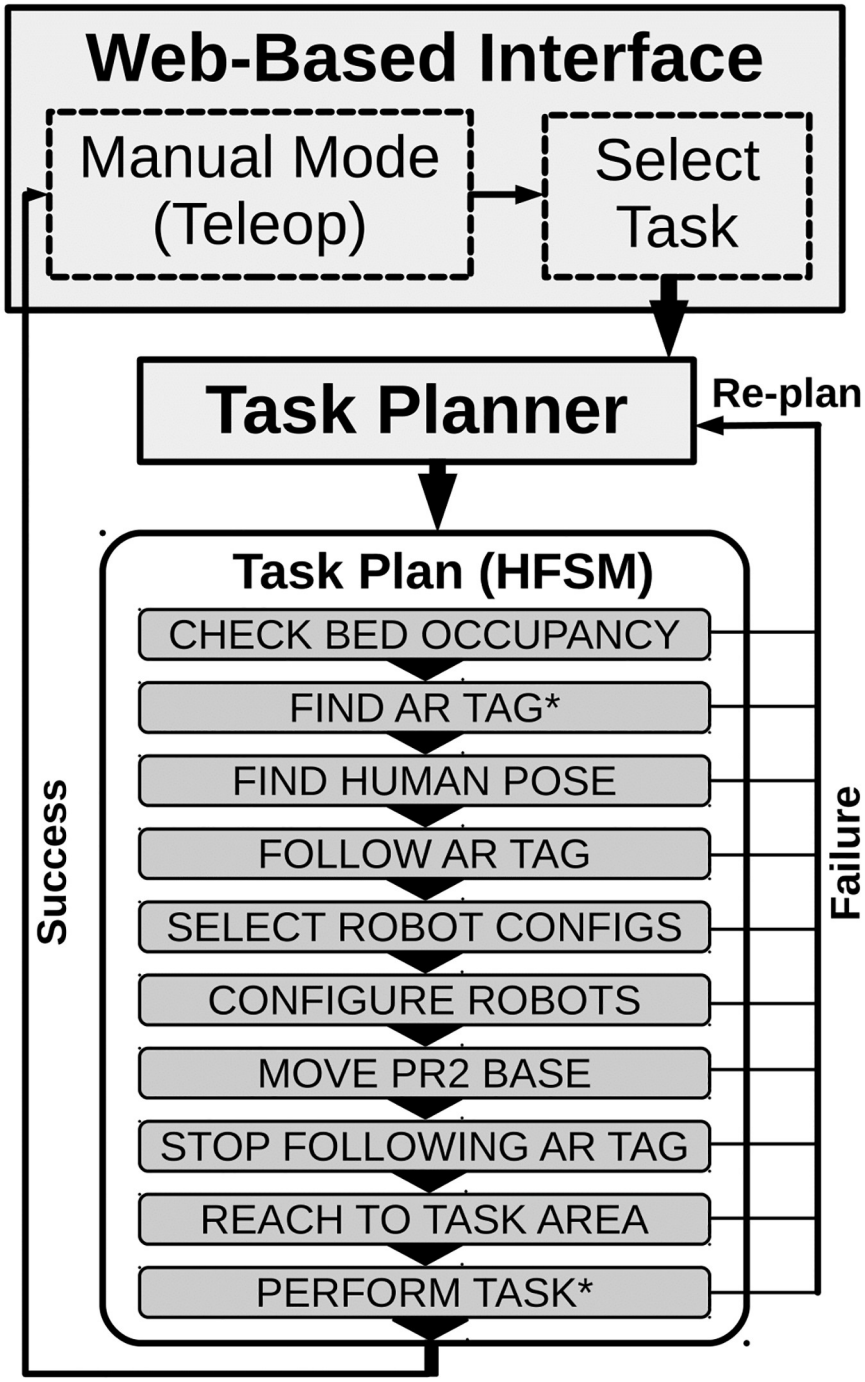
A flowchart showing the process of using the system. From manual mode, the user can either teleoperate the robot or select a task. The task planner generates and then executes a task plan. Each state in the plan either succeeds and continues to the next state, or causes the planner to replan. The plan ends by returning the system to manual control mode. *Indicates a subtask requiring user input.

Fig 6 shows a typical task plan from start to completion of a task. In the state in the plan, CHECK BED OCCUPANCY, the system checks if a person is in the bed. In FIND ARTag, the interface switches into a Looking Mode so the user can move the robot’s head until the Autobed ARTag is in view. In FIND HUMAN POSE, the system checks Autobed’s pose estimate for the user. In FOLLOW ARTag, the PR2 begins to track the ARTag and keep its head pointed at it. In SELECT ROBOT CONFIGS, the system uses TOC to select a configuration for the robots for the task based on the user’s current pose on the bed. In CONFIGURE ROBOTS, Autobed moves to the optimized configuration and the PR2 moves to the optimized height and moves its arms to an initial configuration. In MOVE PR2 BASE, the PR2 uses ARTag servoing to the optimized configuration for its base relative to the Autobed. In STOP FOLLOWING ARTag, the PR2’s head stops tracking the ARTag, returning control to the user. In REACH TO TASK AREA, the PR2 moves its gripper to the task area. In PERFORM TASK, the system switches to the manual Arm Control Mode of the user interface for the user to provide the fine control to complete the task. Each state either succeeds and proceeds to the next state, or fails and causes the system to re-plan.

In our two studies, all 6 tasks followed the same overall task plan described above with differences in their optimized task configurations and task areas for the gripper. For example, the plan for the feeding task used a goal pose for the gripper near the person’s face for the REACH TO TASK AREA step, while the bathing legs task used a goal pose for the gripper near the legs. As the system moves through the plan, it sometimes requires assistance from the user to advance to the next state (e.g. using teleoperation commands to locate the ARTag above the bed, which is then tracked autonomously).

## Evaluation

Within this section, we describe the three evaluations we conducted. Each evaluation addressed a key question relevant to the effectiveness of this type of bedside assistance system. The first evaluation estimated the body pose estimation accuracy of our system. The second evaluation assessed whether collaboration between the robotic bed and the mobile manipulator improved the performance of the overall system. The third evaluation determined whether the bedside assistance system could be successfully used in a real healthcare environment by a person representative of people we expect to benefit from this type of system.

### Evaluation 1: What is the head pose estimation accuracy?

The robotic bed’s estimation of the human’s body pose is a key aspect of the bedside assistance system. The system’s autonomous capabilities make use of a 3D model of the person’s body on the robotic bed. We characterized the performance of the body pose estimation in order to quantify its role. Specifically, our evaluation quantified the head pose estimation error along the width of the bed model (Y-axis).

For this evaluation, there were 8 able-bodied participants who took part in the study between August and December 2015. The participants gave written informed consent (as outlined in the PLOS consent form) to publish the details included in this article. Data from the study can is available at DOI: 10.6084/m9.figshare.9770690. Participants’ weights ranged from 52 to 95 kg and heights from 1.60 to 1.87 m. For the experiment, we placed Autobed in a motion capture room. We asked the participants to lie on Autobed in a supine configuration comfortable to them, keeping their faces pointed straight up from the bed, while wearing infrared reflective marker arrays on their bodies and heads (see Fig 4 Left). The bed was in a flat configuration for this evaluation. We designated the projection of the center of the forehead marker array onto the plane of the bed as the ground truth head position.

We selected 50 pressure distribution images from each participant while they were lying on the bed with their faces pointed straight up from the bed to form our test dataset of 400 pressure distribution images. The images for each participant were collected over a 28 second period on average. We compared the estimated head position with ground truth, but only considered error along the width of the bed (along the Y axis), which the system uses to position the model of the person’s body.

### Evaluation 2: Does robot collaboration improve task performance?

The premise for the system is that a mobile manipulator and robotic bed can collaborate together to provide better assistance. Our second evaluation focused on testing this premise and considered both physical and perceptual collaboration between the two robots. This experiment took place in February 2017. We quantified the performance of the system in terms of how well the mobile manipulator could reach task-relevant poses with its end effector. Robots would be better able to assist with a variety of tasks if they could move objects to locations near a person’s body, such as a utensil to the mouth or a lotion applicator to the arm.

#### Implementation details

In these experiments we used a weighted medical mannequin (∼48 kg). We included into TOC’s environment model a wall behind the bed, which matches our test environment. We roughly modeled the geometries of the bed and expanded the bed model by 4 centimeters to provide a safety margin. TOC searched for up to two configurations for each task on any side of the bed. TOC uses covariance matrix adaptation evolution strategy [74](CMA-ES; implemention from [75]), to perform its optimization search, and we used a population size of 3000 with 10 iterations as the meta-parameters for the optimization for all tasks. When running the optimization to find configurations for situations without physical collaboration between the robots, we decreased the number of parameters to exclude the bed’s degrees of freedom. For one of the tasks, the feeding task, we required that the backrest be at a seated configuration (≥ 55 degrees from horizontal) to emulate a typical eating posture for humans.

Throughout our experiments, the PR2 used its left arm to perform the task and kept its right arm to its side. For some difficult to reach task areas, TOC chose to use two configurations for the task. In these cases, the experimenters ran the system for each configuration separately, starting the PR2 on the appropriate side of Autobed.

#### Experimental protocol

An able-bodied experimenter sitting at a nearby desk used the web-based interface to simulate performing three tasks on the mannequin lying on Autobed. The tasks were feeding, bathing the lower legs, and arm skin care (e.g., applying lotion). These tasks are representative of ADLs, specifically feeding and hygiene. ADLs are important for independent living and quality of life [76–78].

**Fig 7:**
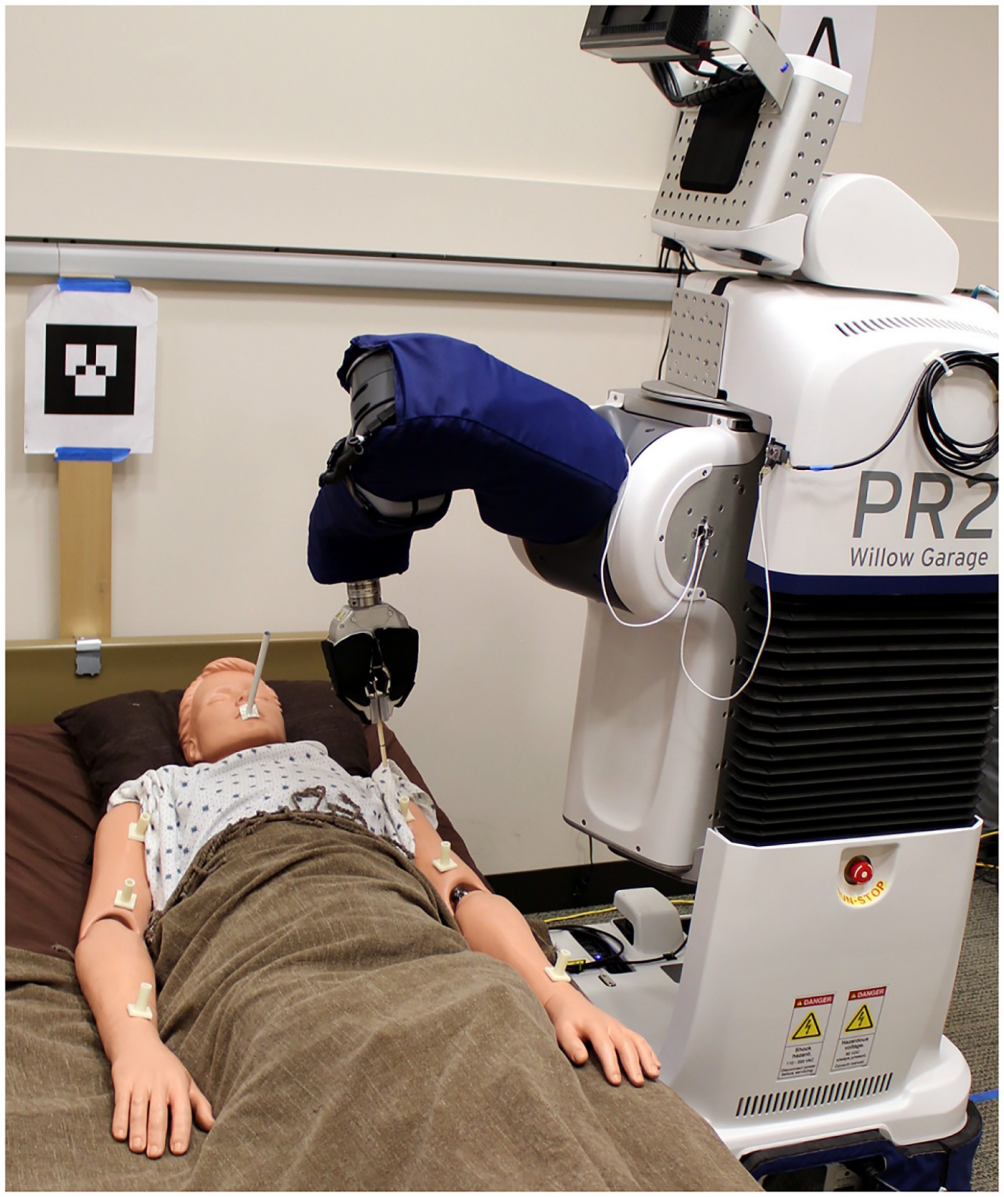
The system after execution of the autonomous functions for the arm hygiene task. The PR2 and Autobed have configured themselves for the task and the PR2 has reached its arm to the task area on the mannequin.

For each trial, we started the PR2 on the same side of the bed as the goal position, ∼1 m away and facing Autobed, and looking at the ARTag mounted on Autobed. Using the web-based interface, the experimenter commanded the system to begin the task. The system then autonomously generated and executed a plan, configuring the PR2 and Autobed for the task, and moving the left end effector to the task area. Fig 7 shows a configuration reached using the system for the *mouth wiping* task with the mannequin on the left side of the bed.

**Fig 8:**
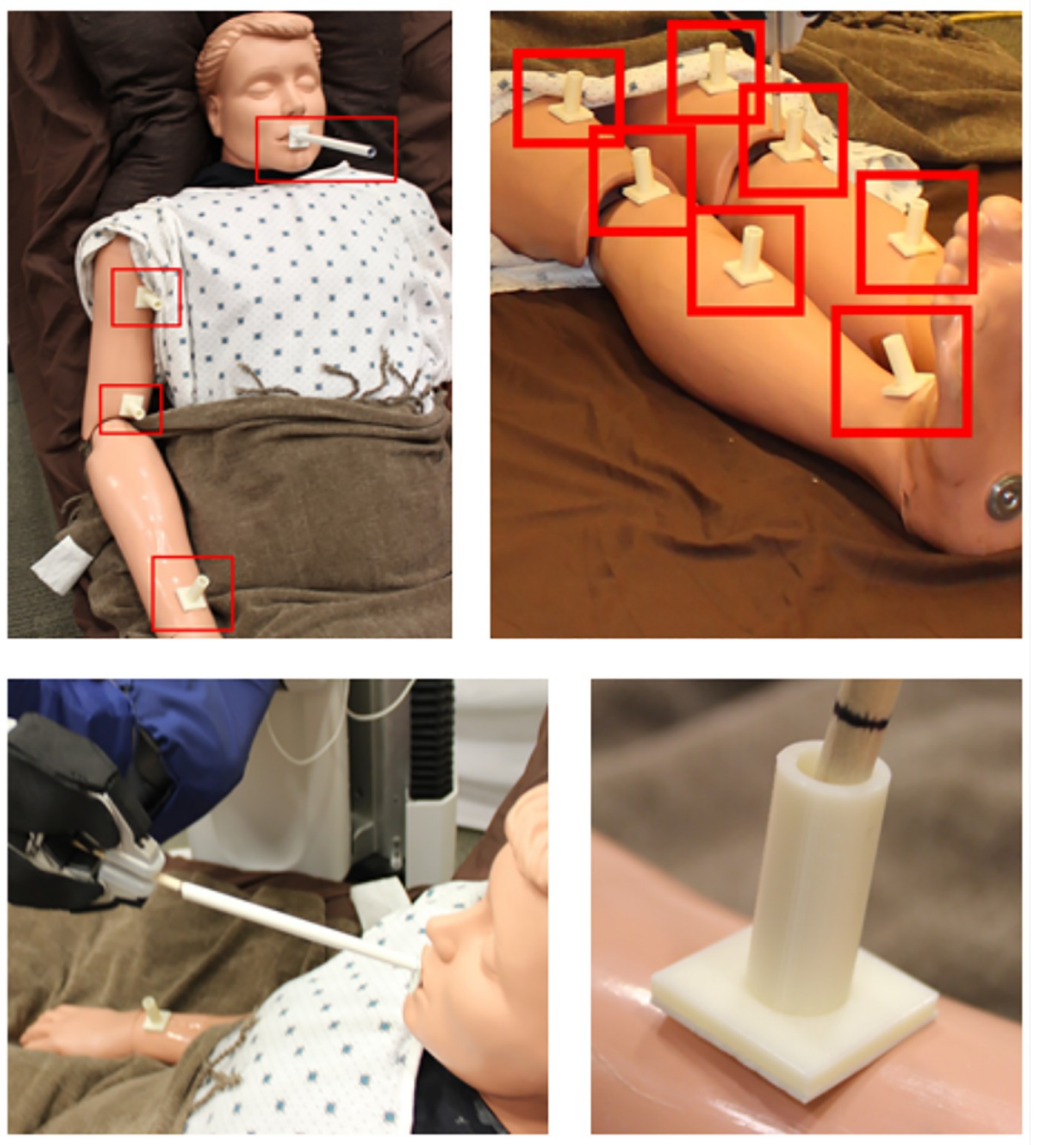
The evaluation of the system with a mannequin. Top: Mannequin with the physical 5-DoF goals highlighted for the feeding task and the right arm of the arm hygiene task (left) and for the leg bathing task (right). The left and right side had symmetric goals. Bottom: Left: Example of the PR2 successfully reaching the goal pose for the feeding task, inserting a 20 cm long cylindrical tool into the 15 cm tube on the mannequin’s mouth. Right: Example of failing to reach a goal pose. The tool could not reach the bottom of the tube without touching the walls of the tube.

At this point in the experiment, the experimenter deactivated the PR2’s motors and manually moved its left end effector to several task-specific goal poses around the task area (8 for bathing legs, 6 for arm hygiene, 1 for feeding). We affixed a hollow tube to the mannequin at each goal location. The PR2 held a small cylindrical tool in its left end effector. If the experimenter could reach the base of the tube with the PR2’s held cylinder without touching the walls of the tube, we considered the PR2 as being able to reach that goal (see Fig 8). The tubes for bathing legs and arm hygiene were 3 cm long. The tube for feeding was 15 cm long. Note that the PR2 has infinite-roll wrists making the goal poses 5-DoF.

For the bathing legs task, the 8 goals poses were along the tops of the legs, ranging from 10 cm above the knee to the ankles. For the arm hygiene task, the 6 goal poses were along the front or top of the arms, ranging from the wrist to the shoulder. For the feeding task, the single goal pose was a attached to the center of the mouth. The goal locations are shown in Fig 8. If the PR2 could reach all goals for a task from the one or two configurations selected by TOC, without further motion of the Autobed or the PR2 mobile base, the system was considered to have succeeded. Otherwise, the system was considered to have failed. Because the goals are sparse and represent a more complicated task, we deemed reaching all goals necessary for success. For the feeding task, the pose of the person’s body matters; it is typically comfortable to eat while seated upright. We required that the backrest be ≥ 55 degrees from horizontal and that the goal be reached for the feeding task to be judged successful.

To evaluate the performance of the system without physical collaboration between the two robots, we ran the experiment with Autobed fixed in its lowest, flattest configuration, which is comparable to a standard bed. TOC was given an unactuated model of the bed in this configuration, so the system could optimize configurations accordingly. To evaluate the performance of the system without perceptual collaboration, we ran the experiment with Autobed always reporting that the mannequin was in the center of the bed.

### Evaluation 3: Can a person with severe motor impairments successfully use the system at home?

We evaluated the system with Henry Evans in the bedroom of his home. Henry is a person with severe motor impairments who requires assistance and spends substantial time in bed. Henry Evans, who frequently collaborates with our lab, used the system in his home to repeatedly perform three assistive tasks around his body. This study took place in February 2017. Henry gave written informed consent (as outlined in PLOS consent form) to publish these case details and use his name. The individuals featured in the attached S1 Video showing part of this evaluation have provided written informed consent (as outlined in PLOS consent form) to publish their image alongside the manuscript. Henry has severe quadriplegia and is mute as the result of a brain-stem stroke. He only has limited movement in his head and left arm and hand, although he has full sensation. Henry regularly uses a computer via a head-tracking mouse from Madentec.

#### Implementation details

We brought a PR2 to Henry Evans’ home and converted his standard electronic bed (Invacare Full-Electric Homecare Bed, product ID: 5410IVC) into Autobed. During late night trials we introduced an additional light in the room to aid in visual perception of the ARTag, but otherwise we did not alter his bedroom. Fig 1 shows Henry’s bedroom, where the experiment took place.

Henry’s particular form of human-computer interface (HCI) requires that he see his laptop screen and the head-tracking device attached to it. We gave TOC a coarse model of Henry’s bedroom and his laptop, as well as virtual obstacles representing locations at which the robot would obstruct Henry’s view of his laptop and the head-tracking devices view of a marker he wears on his glasses. These virtual obstacles were rays forming a cone of vision from the human model’s eyes to all parts of the screen (see Fig 5). TOC used a human model that approximated Henry’s ability to move his head and matched his height. As in the mannequin experiments, we expanded the bed model by 4 centimeters to provide a safety margin and the PR2 used its left arm to perform the tasks, keeping its right arm at its side.

Because only one side of Henry’s bed is accessible, we allowed TOC to optimize only a single configuration for each task, limited its search space to the accessible side of the bed, and required the left arm’s workspace to at least partially overlap the bed. We also limited the optimization of the Autobed’s backrest angle to stay within the range of 40 to 55 degrees from horizontal, so that Henry could comfortably operate the laptop. Because of the constrained region for motion around Henry’s head and safety considerations, the system did not autonomously reach to the task area for tasks around Henry’s head. Instead, the PR2 left its arms in their initialization pose when it initiating the manual control part of the task plan.

#### Experimental protocol

For each trial, we started the PR2 ∼1 meter away from the bed, roughly midway down the bed, with its base facing the bed, its arms to its sides, and its head pointing at the ARTag mounted on Autobed. Autobed started lowered to the ground as far as it could go with its backrest at 45 degrees and leg rest down. Henry started each trial lying on the bed; we did not alter his position from where his caretaker lay him. Henry controlled the system using a laptop on an overbed tray.

For each task, Henry was required to use the system’s autonomous function associated with that task and then only use the interface’s left arm control to perform the task. Henry provided high-level oversight of the system, including selecting the task, stopping the system if something goes wrong, moving the robot’s head to see the ARTag if necessary, and teleoperating the robot’s left arm after the autonomous mode has transferred control to the to him. The system used TOC to select one configuration for the PR2 and Autobed for the task, based on the estimated position of Henry’s body. Henry made use of his own physical capabilities to assist in task performance. For example, for the tasks around his head, Henry would move the towel held in the PR2’s gripper to an appropriate location and then wipe his face on the towel by moving his head.

The tasks performed were:

(a) Pulling a blanket down from knees to feet
(b) Wiping yogurt from mouth
(c) Wiping lotion from forehead

Previous work has noted that these three tasks may be useful for those with severe motor impairments [24] and Henry has indicated that these tasks would be useful.

For each task, Henry was given instructions on how to use the interface and was given practice with the system until he felt comfortable. He then performed the task 5 consecutively. An experimenter set up each task’s trials by a) covering the feet and knees with a blanket; b) applying yogurt to both sides of Henry’s mouth; or c) applying dabs of lotion to the center of Henry’s forehead. For the wiping tasks, a fresh, rolled towel was placed in the PR2’s left gripper before each performance of the mouth wiping task. The goal of each task was explained to Henry, and he decided if a task was completed successfully. He used his laptop’s camera to inspect his face to check for yogurt and lotion and used the PR2’s camera to inspect the state of the blanket. Trials were considered to have failed if any interruptions in the system’s performance, such as an experimenter intervention, occurred, or if Henry deemed that manual movement of the PR2 mobile base or Autobed was necessary to complete the task. For each trial, we recorded success (or failure), the time to complete the task, and solicited free-form feedback from Henry.

## Results

Within this section, we present the results from our three evaluations.

### Head-pose estimation error was sufficiently small

The head pose estimation error along the width of the bed (i.e., the Y direction) was 5.00 cm ± 2.54 cm (mean ± std). Our previous work in simulation suggested that task success could be achieved with this error by using TOC to select robot configurations [3]. We show evidence in our other evaluations that body pose estimation performance improved the overall system performance in a laboratory setting and was sufficient to function in a real-world setting.

### Robot collaboration improved task performance

Table 1 shows overall performance and Table 2 shows the task outcome and number of reached goal poses for each task as we translated the mannequin from the center of the bed in the Y direction by −15, 0, and 15 cm. Using all parts of the system, the PR2 was successful in 100% of the tasks, reaching all goals.

**Table 1:** Overall performance, percentage of successful tasks by the system with and without bed movement or human position estimation.

*Condition*	*Overall Performance*
Full System	**100**% (9/9)
No Bed Movement	**33**% (3/9)
No Position Estimation	**78**% (7/9)

**Table 2:** System performance with and without physical or perceptual collaboration. Task is successful if all goals reached.

		Full System		No Bed Movement		No Position Estimation	
*Task*	*Body Shift*	*Outcome*	*Poses Reached*	*Outcome*	*Poses Reached*	*Outcome*	*Poses Reached*
Feeding	-15 cm	success	100% (1/1)	failure	N/A	success	100% (1/1)
	0 cm	success	100% (1/1)	failure	N/A	success	100% (1/1)
	15 cm	success	100% (1/1)	failure	N/A	success	100% (1/1)
Arm Skin Care	-15 cm	success	100% (6/6)	failure	83% (5/6)	success	100% (6/6)
	0 cm	success	100% (6/6)	failure	50% (3/6)	success	100% (6/6)
	15 cm	success	100% (6/6)	failure	83% (5/6)	failure	83% (5/6)
Bathing Legs	-15 cm	success	100% (8/8)	success	100% (8/8)	failure	88% (7/8)
	0 cm	success	100% (8/8)	success	100% (8/8)	success	100% (8/8)
	15 cm	success	100% (8/8)	success	100% (8/8)	success	100% (8/8)

#### Physical collaboration improved task performance

**Fig 9:**
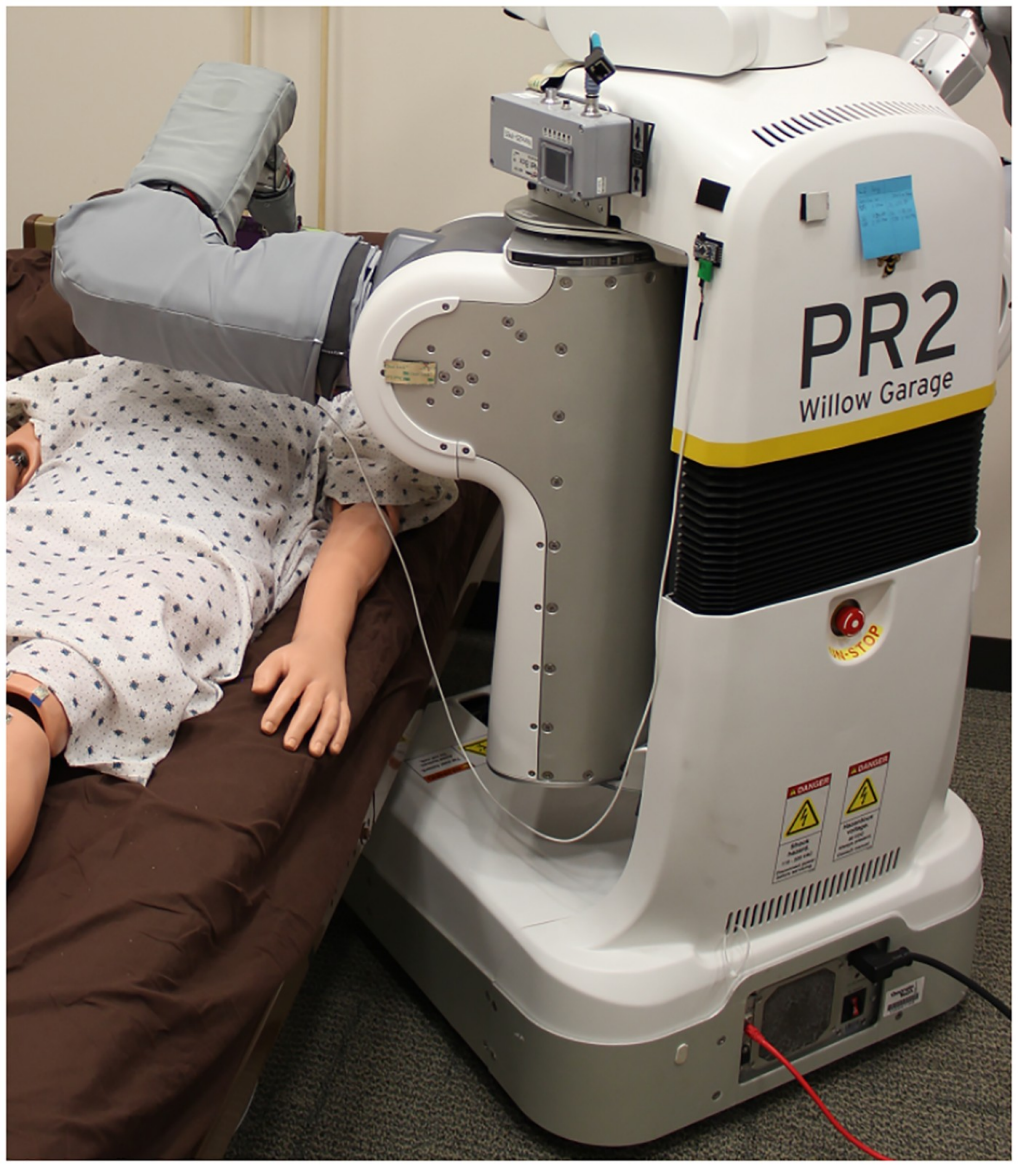
Physical collaboration between the two robots. A solution frequently used by the system was to raise the bed and to move the PR2’s base under it, so the PR2 could better reach task areas.

The two robots collaborated physically to allow the PR2 to better perform the task. Changes to the Autobed configuration gave the PR2 better physical access around the mannequin and adjusted the mannequin’s configuration for the task. For example, Fig 9 shows a solution frequently used by the system, which was to raise the bed and to move the PR2’s base under it. Without physical collaboration between the two robots, the system succeeded in 33% (3/9) of the tasks. In the feeding task, Autobed was needed to achieve the task goal of having the person sitting upright (≥ 55 degrees from horizontal). When the system could not move the bed, it could not succeed at this task.

#### Perceptual collaboration improved task performance

The robots collaborated perceptually to estimate the mannequin’s position and configuration, allowing better initial configuration selection for the task, and also to inform the PR2 when autonomously moving its end effector to the task area. Without perceptual collaboration between the robots, the PR2 succeeded in 78% of the tasks, since it was sometimes too far from the mannequin to reach the goal poses.

### A person with severe motor impairments successfully used the system in his home

**Fig 10:**
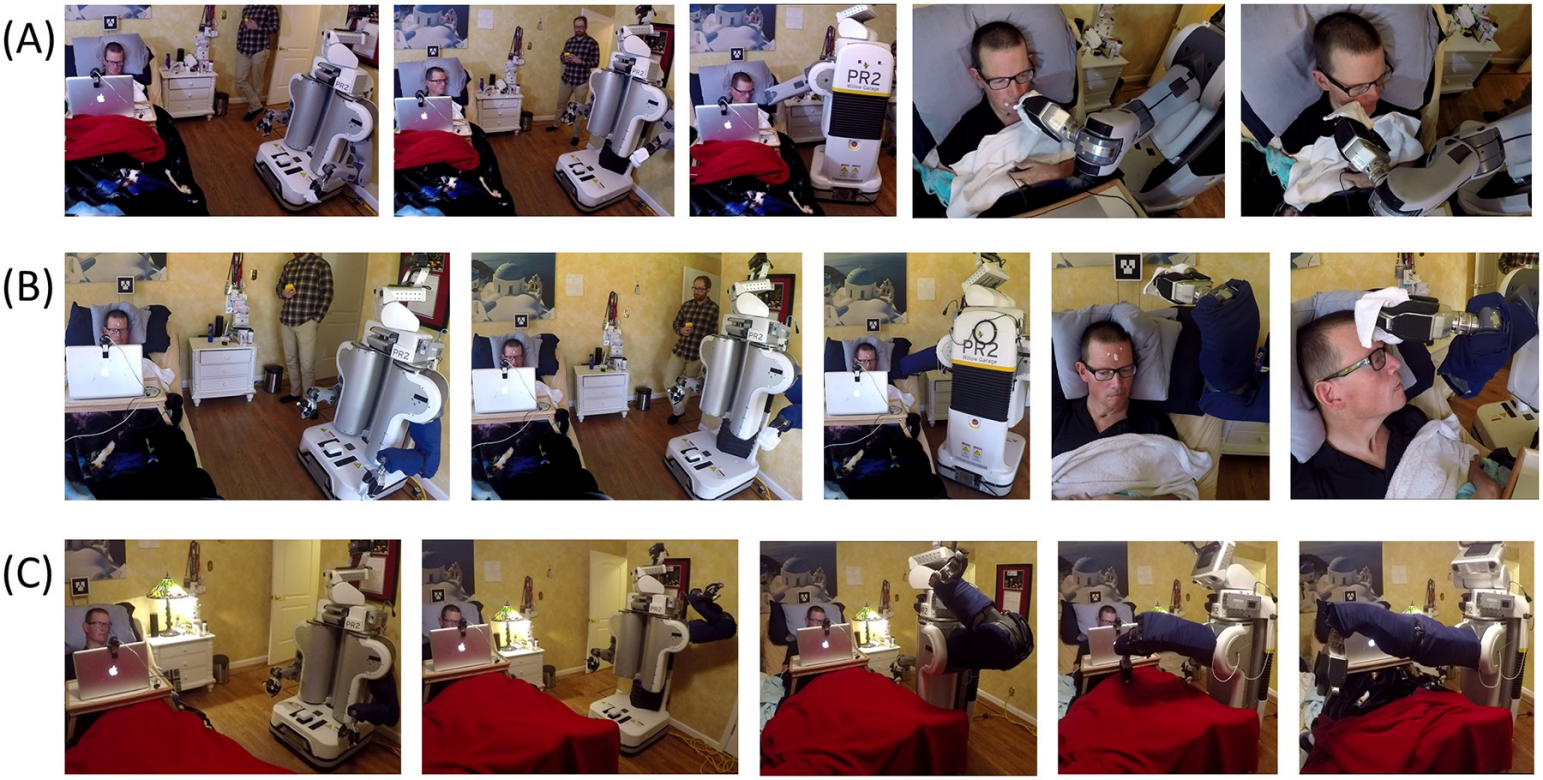
Image sequences from the three tasks performed by Henry. Using this system, Henry Evans was able to (A) wipe yogurt from his mouth, (B) wipe lotion from his forehead, and (C) pull a blanket down from his knees to his feet.

Fig 10 shows sequences of images of the performance of the three tasks by the system. Table 3 shows the percentage of successful trials and times for each trial. It took 3 minutes and 33 seconds on average to complete each trial across all tasks and he was successful in all trials. The errors we were collecting were if the task could not be completed without moving the mobile manipulator’s base or reconfiguring the bed, or if Henry lost control of the robot and could otherwise not complete the task (for example, if the robot blocked his view of the laptop and he could no longer control the robot). No such errors were observed during the tests. During the blanket adjustment task, Henry said “It works well and is very easy” and “Almost no mental effort. That’s how I like it. This would be great for a first time user.” After the fourth trial of the forehead wiping task, Henry said “It required a lot of mental effort at first, but now it’s easy.’ After the last mouth wiping task, Henry said “Most complicated task yet.” Wiping the yogurt from the mouth seemed particularly challenging because wipes tended to smear yogurt around his face. During the autonomous portion of each task, Henry exerted little effort, simply watching the task plan execute. He seemed to particularly like that the robot autonomously reached to the task area in the blanket task.

**Table 3:** Performance of our system used by a person with severe quadriplegia in his own home to perform three real tasks. Movement of the PR2 mobile base and of Autobed was performed only by the autonomous portion of the system.

Task	% Trials Successful	Time per trial, in order (min:sec)				
Wipe mouth	100% (5/5)	5:30	3:59	4:31	3:13	3:33
Wipe forehead	100% (5/5)	3:23	3:44	4:18	3:18	2:25
Adjust blanket	100% (5/5)	3:23	3:00	3:10	3:01	2:39

Henry noted that he found the optimized robot configurations non-obvious for the tasks around the head, but he recognized their value after using them. From those configurations he noted that the robot could reach the goal areas surprisingly well without colliding with obstacles or blocking his view of the laptop.

## Discussion

We sought to fairly handle the comparison between the full system and the system without allowed bed movement by using the same parameters (e.g., search time) for the optimization. However, because of the nature of the problem, a non-convex optimization in a large search space, searching longer may have found better configurations than we used in our experiments.

We found that our system was capable of reaching many or all goal poses even with 15 cm of error in human position estimation. This was particularly noticeable in the feeding task, where the system without position estimation succeeded in all tasks. Because Autobed is narrow, we could not feasibly position the mannequin more than 15 cm from the center of Autobed. We would expect estimation of the person’s pose to be more important in other environments, such as wider beds and beds with nearby obstacles that restrict access.

Our evaluations demonstrated that a geometric and kinematic model of the human body with a simple pose estimation method can result in an effective bedside assistance system. We expect that higher-fidelity human body models and pose estimation methods, such as from [57], would further improve the system’s performance and create new opportunities for assistance. Comparable performance to our current system might also be achievable with less costly sensors instead of a full-sized pressure-sensing mat.

In our previous research on the basic teleoperation system for the PR2 robot, slow speed of operation and high error rates were notable areas for improvement [28]. The autonomy in our bedside-assistance system may help address these issues. Henry Evans provided feedback throughout the development of the bedside assistance system, including testing earlier versions. Due to his experience teleoperating the PR2 and the bedside assistance system, we chose not to formally compare his performance with and without autonomy.

In preliminary tests with earlier versions of the system, Henry’s first attempt at a task without autonomy would sometimes take a long time. However, with several repetitions of the task, Henry could improve his time. For example, for a task of touching his left knee with the robot, he improved from 6 minutes to 2 minutes to complete the task. This potential for improvement with practice may be a relevant alternative to autonomy, although, even with Henry’s expertise, teleoperating the robot appeared to require his full attention. In addition, he appeared to be intensely focused on performing tasks quickly when he achieved his shortest times.

We observed that when using teleoperation methods that we have previously developed, Henry would sometimes experience errors that would result in loss of control of the robot. Most commonly, when he attempted a task around his face, Henry would move the arm of the robot so that it obstructed his laptop or head-tracker, and he would lose the ability to control the robot via his laptop. We observed no errors when Henry used the autonomous bedside-assistance system.

## Conclusion

We have presented a robotic system designed to provide physical assistance to people who are in bed, increasing user independence and reducing caregiver burden. The system consists of a robotic bed and a mobile manipulator that collaborate to assist with tasks around the user’s body. The robotic bed senses the pose of the human body using a pressure sensing mat and then the robotic bed and the mobile manipulator move to configurations that have been optimized for the task. After achieving the optimized configurations, control can be transferred to the user immediately or after the mobile manipulator has reached autonomously toward a task-relevant location.

Our three evaluations provide evidence for the effectiveness of our bedside assistance system. The first evaluation helped show that the body-pose estimation error is sufficiently small to be useful. The second evaluation found that physical and perceptual collaboration between the mobile manipulator and the robotic bed improved the system’s performance. The third evaluation demonstrated that the system can be successfully used at home by a person with severe motor impairments who spends time in his bed throughout the day. Overall, our results suggest that a robotic bed and a mobile manipulator can work collaboratively to provide effective personal assistance and that the combination of the two robots is beneficial.

## Supporting information

S1 Video A video demonstrating the evaluations of our system for bedside assistance that integrates a robotic bed and a mobile manipulator. The individuals featured in the video have provided written informed consent (as outlined in PLOS consent form) to publish their image alongside the manuscript.
(MP4)
